# Focal Cemento Osseous Dysplasia: A Case Report

**DOI:** 10.30476/DENTJODS.2022.88067.1309

**Published:** 2022-06

**Authors:** Safoura Seifi, Hakimeh Ghorbani, Oveis Khakbaz, Fatima Bijani

**Affiliations:** 1 Dept. of Oral and Maxillofacial Pathology, Oral Health Research Center, Health Research Institute, Dentistry Faculty, Babol University of Medical Sciences, Babol, Iran; 2 Dept. of Oral and Maxillofacial Radiology, Oral Health Research Center, Health Research Institute, Dentistry faculty, Babol University of Medical Sciences, Babol, Iran; 3 Dept. of Oral and Maxillofacial Surgery, Oral Health Research Center, Health Research Institute, Dentistry faculty, Babol University of Medical Sciences, Babol, Iran

**Keywords:** Fibro osseous lesions, Focal Cemento Osseous Dysplasia, Cemento Ossifying Fibroma

## Abstract

Focal cemento osseous dysplasia (FCOD) is a subgroup of benign fibro osseous lesions, which occur in a single site of tooth-bearing areas of jaws. It is usually asymptomatic and noticed accidentally through routine radiological exams. There is often no need for treatment of such lesions. This case was a 28-year-old male patient with a mixed radiolucent-radiopaque lesion in posterior part of his mandible. Based on radiographic examination, cemento ossifying fibroma (COF) was the first differential diagnosis. After the surgical removal of the lesion, histopathologic evaluation was made and the case was diagnosed as FCOD. Fortunately, after a few months of operation, there was no complication and complete bone formation was occurred.

## Introduction

Cemento osseous dysplasia occurs in the tooth-bearing areas of jaws and is a subgroup of benign fibro osseous lesions of jaws. The precise cause is unknown, but periodontal ligament is suggested as a probable origin [ 1 ]. Based on clinical and radiographic features, WHO subdivided it into three groups including focal, periapical, and florid [ 2 ].

Focal cemento osseous dysplasia (FCOD) involves a single site most commonly posterior of mandible. Before the concept of FCOD was clarified in the mid-1990s, most cases were misdiagnosed as a variant of ossifying fibroma [ 1 ]. The most affected patients are African or Southern Asian women and the age of diagnosis is between 30-50 years [ 3 ]. Cemento osseous dysplasia is often asymptomatic and the adjacent teeth are vital. Therefore, it is usually discovered on routine radiographic examination [ 1 , 4 ]. 

Initially in osteolytic stage, it appears as a radiolucent area. Sometimes it is misdiagnosed as dental periapical lesions and unnecessary extraction or root canal treatment is done for patients. While the condition progresses, the radiographic pattern changes into mixed because of bone repair through the defect. Finally, at the mature stage, the lesion develops to a radiopaque image with a rim of radiolucency. The borders are well defined and slightly irregular [ 3 - 4 ].

Histologically, fragments of cellular fibrous connective tissue with scattered bony trabeculae are seen and hemorrhage around them is a common finding.

Diagnosis of FCOD can be established by clinical and radiographic findings; however, in some cases, biopsy is crucial to rule out some other lesions such as ossifying fibroma. In surgical aspect, ossifying fibroma tends to separate easily from its surrounding bone while cemento osseous dysplasia consists of gritty tissue in small fragments [ 1 ].

Regarding the nature of this lesion, there is no need for treatment [ 5 ]. In asymptomatic lesions, regular follow up examinations are recommended [ 6 ]. Overall, the prognosis is good [ 1 ].In a practical approach towards the definitive diagnosis of benign fibro-osseous lesions, it is important to analyze all correlating factors in a case. The demographics, clinical history, intraoperative appearance, macroscopic view of gross specimen, and most importantly, the microscopic (histopathologic) and radiographic evaluation are crucial for an accurate diagnosis. Pathologist’s judgment is still the most important point to make a definitive diagnosis that will result in the appropriate treatment [ 7 ].

In this study, we discuss about a case of FCOD in posterior part of mandible, which could not be differentiated accurately from ossifying fibroma by radiological exams.

## Case Presentation

**Figure 1:**
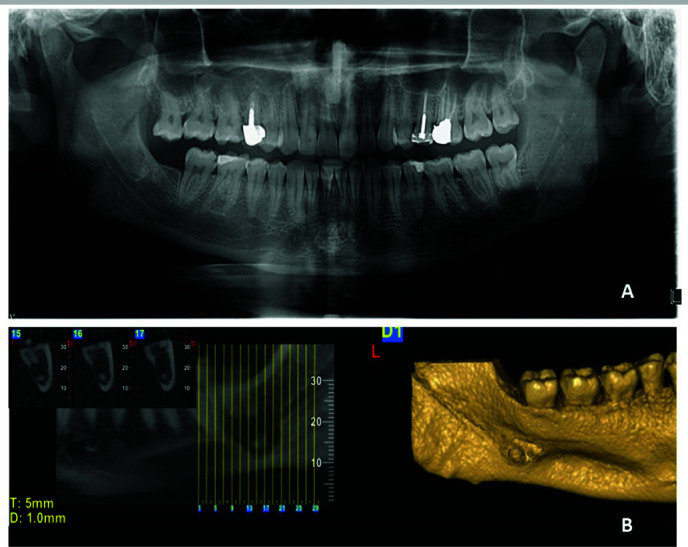
**a:** Panoramic view of patient with a well-defined mixed radiolucent-radiopaque lesion next to the apex of left mandibular third molar, **b:** CBCT showed expansion and thinning of lingual cortical plate in cross-section view

**Figure 2:**
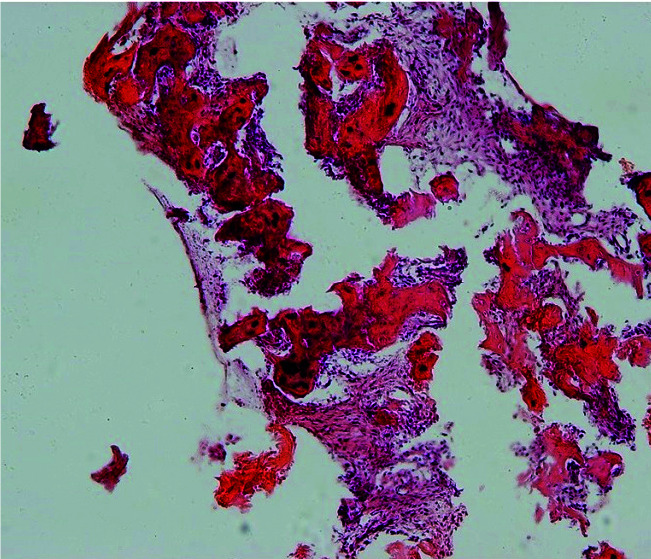
Microscopic evaluation showed accumulation of hyper cellular and fibro vascular connective tissue with scattered hemorrhage and mixture of woven and trabecular bone and cementum like particles. The particles had irregular borders with retraction from adjacent stroma. (H&E 100 x)

At May 2018, a 28-year-old male patient was referred to private clinic of maxillofacial surgery after his dentist had noticed a round well-defined mixed radiolucent-radiopaque lesion next to the apex of left mandibular third molar. There were no signs of root resorption or tooth displacement (Figure 1a). Clinical examination did not show any abnormality such as periodontal pocket or ulcer and inflammation in the surrounding gingiva. The involved tooth was also vital. Cone beam computed tomography (CBCT) was recommended for the patient, which showed expansion and thinning of lingual cortical plate in cross-section view (Figure 1b). The lesion did not involve the mandibular canal. Based on these findings, the first differential diagnosis of radiologist was cemento ossifying fibroma (COF). Therefore, the surgeon determined to excise the lesion after extraction of the related tooth. The lesion was hemorrhagic and separated from surrounding bone in small spongiotic fragments. A small particle of hard tissue was found in the center of the defect. Since the lesion could not been removed from adjacent bone easily and due to hemorrhagic nature of the lesion, the diagnosis was tended to be cemento osseous dysplasia. The excised tissues were placed in formalin and sent to laboratory for histopathologic evaluation. The histopathologic evaluation showed accumulation of hyper cellular and fibro vascular connective tissue with scattered hemorrhage and mixture of woven and trabecular bone and cementum-like particles. The particles had irregular borders with retraction from adjacent stroma. Globular mass in the center of lesion consisted of thick and connected trabeculae (Figure 2).

**Figure 3:**
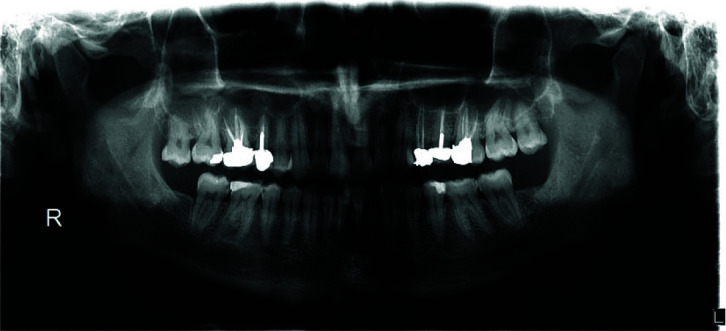
Panoramic view of patient 6 months after operation showed complete bone reconstruction without any abnormality

According to these clinical, radiographic and histopathologic findings, the final diagnosis was FCOD. Based on literature, there was no need for any other interference and patient was under regular follow up. After 6 months of operation, the clinical evaluation showed total healing and no sign of abnormality. In panoramic view, complete bone reconstruction was evident (Figure 3).

## Discussion

Cemento osseous dysplasia is an asymptomatic lesion, which is often detected in routine radiographic examination. If the lesion is found in just one area of jaws, especially in the posterior part of mandible, the term FCOD is used. This lesion is common in African women and has three stages of developing usually diagnosed at mean age of 41 [ 1 ]. This patient was a young Middle Eastern man and according to radiographic pattern of the lesion, it was compatible with the early stage of cemento osseous dysplasia.

Cemento osseous dysplasia is mainly diagnosed with radiographic examination and there is no need for biopsy or other interferences. Regarding the literature, expansion and thinning the cortical plates are relatively common findings in periapical and florid cemento osseous dysplasia, but in focal cemento osseous dysplasia, it is a rare condition [ 4 ]. In this case, radiographic data showed special pattern of radiolucency with expansion and thinning of the lingual cortical plate. In fact, after early diagnosis of COF, excision of the lesion seemed mandatory. According to the literature, any interference such as surgical excision into cemento osseous dysplasia can frequently lead to bone infection or osteomyelitis, especially after tooth extraction in mandible, and since the management of such lesions is challenging [ 8 ], clinicians prefer to follow these patients rather than surgical interference. However, in this patient, complete healing without any complication occurred.

As Nelson BL *et al*. [ 7 ] believed that definite diagnosis of fibro osseous lesions is based on pathologist’s judgment; the final diagnosis of this case was made according to microscopic features. Although the radiologic first differential diagnosis was consistent with ossifying fibroma, the hemorrhagic nature of the lesion through operation led the surgeon to cemento osseous dysplasia and histopathologic evaluation confirmed the diagnosis.

Informed consent was obtained from the patient for his clinical and radiographic evaluation as well as operation. There is no document in this article, which shows patient’s personal information.

## Conclusion

It is very important for dentists and oral surgeons to be familiar with different bony lesions. Early detection of abnormalities hinders the progression of disease and subsequent dysfunction.
